# A call to action: dissemination and implementation science education and training is needed in physical therapy and athletic training health professions

**DOI:** 10.3389/fmed.2025.1677517

**Published:** 2025-12-04

**Authors:** Sarah J. de la Motte, Carolyn E. Dartt, Hayley J. Root, Johna Register-Mihalik, Oluwatoyosi B. A. Owoeye

**Affiliations:** 1 MissionReady Prevention Systems, LLC, Carbondale, CO, United States; 2 Consortium for Health and Military Performance, Department of Military and Emergency Medicine, F. Edward Hébert School of Medicine, Uniformed Services University, Bethesda, MD, United States; 3 Henry M. Jackson Foundation for the Advancement of Military Medicine, Inc., Bethesda, MD, United States; 4 Concussion, Learning, and Implementation Science in Athletic Training Lab, Department of Physical Therapy and Athletic Training, College of Health and Human Services, Northern Arizona University, Phoenix, AZ, United States; 5 Matthew Gfeller Center and STAR Heel Performance Laboratory, Department of Exercise and Sport Science, University of North Carolina at Chapel Hill, Chapel Hill, NC, United States; 6 Translational Injury Prevention Lab, Department of Physical Therapy and Athletic Training, Doisy College of Health Sciences, Saint Louis University, St. Louis, MO, United States

**Keywords:** dissemination, implementation, allied health, health professions, athletic training, physical therapy, knowledge translation, evidence-based interventions

## Abstract

Prevention and appropriate management of musculoskeletal (MSK) conditions is a key contributor to a healthy and well population. Physical therapists (PTs) and athletic trainers (ATs) are allied health professionals at the forefront of prevention and management of MSK conditions, working in wide-ranging healthcare settings, from outpatient clinics, with specialized populations (e.g., athletes), within schools, and in industrial settings and public health. PTs and ATs receive extensive education in evidence-based practice for the prevention and treatment of common MSK conditions, but little to no formal education in dissemination and implementation (D&I) science. This curricular omission may contribute to persistent challenges and gaps in the real-world translation of injury prevention and rehabilitation research into sustainable and effective PT and AT practice. Here the authors, five MSK injury prevention and management researchers and clinicians with experience or expertise in D&I science, present a brief background on the emergence of D&I in the MSK literature, and outline short- and longer-term goals for the integration of D&I into PT and AT education and training, setting forth a vision for the prominence of D&I in the PT/AT professions.

## Introduction

Physical therapists (PTs) and athletic trainers (ATs) are allied health professionals at the forefront of prevention and management of musculoskeletal (MSK) conditions, a key contributor to a healthy and well population. PTs and ATs work in wide-ranging healthcare settings, including in outpatient clinics, with specialized populations (e.g., athletes), within schools, and in industrial settings and public health. PTs and ATs receive extensive education in evidence-based practice (EBP) for the prevention and treatment of common MSK conditions according to each professions’ accreditation standards - however, few, if any programs, also address Dissemination and Implementation (D&I) science theories, frameworks, and approaches that integrate concepts across multiple disciplines. A review of accreditation standards from the Commission on Accreditation of Athletic Training Education (CAATE) (1) and the Commission on Accreditation in Physical Therapy Education (CAPTE) (2) reveals a primary focus on EBP and interprofessional education (IPE), along with healthcare administration and quality improvement—but no mention of D&I in any standard, glossary or references. Frameworks such as Reach, Evaluation, Adoption, Implementation, and Maintenance (RE-AIM), the Consolidated Framework for Implementation Research (CFIR), or Theoretical Domains Framework (TDF) are not referenced, and implementation science is not listed among required curricular elements. A broad review of publicly available syllabi and program descriptions from numerous U.S. institutions—including several that have D&I offerings available in other programs and centers—reveals no explicit coursework, competencies, or frameworks related to D&I science in Master of Athletic Training (MAT), Master of Science in Athletic Training (MSAT), Doctor of Athletic Training (DAT), or Doctor of Physical Therapy (DPT) curricula (Table 1).

**Table 1:** Curricular overview of D&I offerings at representative U.S. universities offering professional athletic training and physical therapy degrees*.

University	AT program	PT program	Notes
High Point University	MSAT (3)	DPT (4)	No D&I coursework in core curriculum
University of Florida	DAT (5)	DPT (6)	D&I offered via CE and CTSI (7), not embedded in either curriculum
Ohio State University	MAT (8)	DPT (9)	D&I siloed in CTSI (10); not part of AT or PT core training
Idaho State University	MSAT (11)	DPT (12)	No embedded D&I in core curriculum
University of Iowa	MAT (13)	DPT (14)	No D&I coursework in either program
California State University—Long Beach	MSAT (15)	DPT (16)	No D&I coursework listed in AT or PT curricula
University of Pittsburgh	MSAT (17)	DPT (18)	D&I offered via ICRE (19) and CTSI (20), not embedded in PT curriculum
Eastern Washington State University	MSAT (21)	DPT (22)	No D&I coursework in AT or PT curricula

*MSAT, Master of Science in Athletic Training; DPT, Doctor of Physical Therapy; DAT, Doctor of Athletic Training; MAT, Masters in Athletic Training; CE, Continuing Education; CTSI, Clinical and Translational Science Institute; AT, Athletic Trainer; PT, Physical Therapist; ICRE, Institute for Clinical Research Education.

This curricular omission may contribute to persistent challenges and gaps in the real-world translation of injury prevention and rehabilitation research into sustainable and effective PT and AT practice. This article serves as a call to action for increased D&I science awareness, education, and training for PT and AT health professions and PT/AT researchers and clinician-scientists. Here the authors, five MSK injury prevention and management researchers and clinicians with experience or expertise in D&I science, present a brief background on the emergence of D&I in the MSK literature, and outline short- and longer-term goals for the integration of D&I into PT and AT education and training, setting forth a vision for the prominence of D&I in the PT/AT professions.

## Emergence of D&I literature and its importance for MSK prevention

Scientific literature on D&I for sports injury/MSK prevention began to appear in the early 2000’s, with seminal papers on implementation and implementation research for sports injury prevention programming spearheaded by Donaldson and Finch (23) and Finch (24). The crucial messages about the continual research-to-practice gaps in sports injury prevention began to individually shape each authors’ educational path and line of research at that time. For example, as a postdoctoral fellow, the lead author assisted on a research study aiming to implement and evaluate an MSK prevention warm-up in a pre-deploying military setting and on a compressed timeframe. Despite strenuous and concerted efforts, the study team was ultimately unable to evaluate the warm-up due to poor fidelity and poor compliance, leaving a crucial piece of the project unaccomplished. Ultimately coming to realize the study team lacked sufficient knowledge of D&I to facilitate adequate adaptation and adoption of the warm-up, the lead author intentionally set out to develop a new D&I-focused research agenda in the years to come. Other authors on this paper have had similar experiences, each seeing the need for D&I and D&I research in the military, youth sports, occupational health, and clinical settings. Collectively drawing on these experiences and taking inspiration from Finch and colleagues’ work to improve real-world MSK prevention application, (25) and other relevant D&I work to improve implementation and outcomes in sports medicine more broadly, (26–28) we now provide a collective call for the PT and AT professions to embrace D&I education and training. PTs and ATs operate within socioecological systems, serving as principal actors in the D&I of evidence-based care delivered to their patients/clients. The integration of specific D&I instruction for PT and AT health professions education and research will equip practitioners to address the real-world complexities of preventing and treating MSK conditions and prepare a future generation of leaders in these fields. Acknowledging D&I as an essential and imperative aspect to ensure the translation of best research evidence into clinical practice, we advocate for educating (impacting knowledge) and training (impacting skills) PTs and ATs regarding the foundational principles of D&I science to maximize outcomes in the evidence-based prevention and management of MSK conditions.

## Call to immediate action

### Integrate D&I Science into physical therapy and athletic training health professions education curriculum

D&I science should be formally introduced into educational curriculum for PT and AT health professions studies, particularly highlighting and addressing the persistent research-to-practice gap. D&I terminologies and basic D&I concepts in knowledge translation should be introduced alongside existing curriculum on EBP, with additional consideration for integration with IPE and Learning Health Systems (LHS) curricula that correspond to CAPTE & CAATE standards. A first step in this integration is to familiarize learners with understanding the importance of context alongside content. The incorporation of D&I here can optimize the integration of evidence-based therapies and interventions into routine clinical care across a variety of settings and contexts. Ultimately D&I is way to approach making patient-centered care as feasible, effective, and cost-efficient as possible, supporting both patient and provider goals, concepts that should be viewed positively.

### Expand physical therapy and athletic training post-graduate training to include D&I Science

Including formal D&I training for PT and AT post-graduate students would be helpful to ensure they are equipped to evaluate effectiveness, contextual barriers, and scalability of interventions, all of which are critical for translating research into practice (29). Specific courses related to advanced topics in D&I science and practice should be considered at this level, including theories, models and frameworks in D&I science and practice, hybrid research approaches, implementation at the individual and community levels, and planning for implementation and sustainability in community health. D&I frameworks like RE-AIM and CFIR can be used to evaluate the reach, adoption, and sustainability of interventions. For research components, integrating D&I hybrid approaches into traditional research designs holds the potential to speed up knowledge translation in the MSK field and should be prioritized in PT and AT post-graduate studies accordingly. Providing tools for future researchers and educators can be useful to systematically approach issues that have multi-level challenges and stakeholders involved.

## Call to intermediate action

### Update continuing education and professional development with D&I offerings

Continuing education (CE) and professional development in D&I for PTs and ATs may take many forms and will need to be coordinated with governing/licensing bodies such as the Board of Certification for ATs and CAPTE for PTs. A range of offerings, such as individual study courses for CE credits/units, dedicated D&I tracks within quality improvement/quality assurance pathways for CE requirements, and D&I workshops and learning labs at professional conferences can enhance D&I integration within the PT and AT professions. While entry-level education focuses on foundational content and equips students with skills that improve processes and outcomes on a more local scope, dedicated D&I CE and professional development can build off these experiences and knowledge to include knowledge application to a broader community with testable hypotheses. Essential offerings may include refresher courses on D&I principles and concepts, with the potential to serve as a catalyst for more individualized and in-depth selections depending on individual PT/AT settings and/or professional needs. For example, offerings for clinicians that explore how to utilize D&I principles in real-world scenarios for their setting to improve patient outcomes or efficiency in their own clinical practice would be particularly appealing. Additionally, case study offerings where D&I concepts and frameworks are utilized, improving learners’ utilization of D&I specific to their practice setting would also be practical. For PT and AT researchers and clinician scientists, offerings that provide instruction on new approaches in clinical trial designs where they simultaneously evaluate intervention effectiveness and best D&I strategies for the same intervention they are testing would be beneficial (30).

For those interested in even more in-depth options, a D&I track or pathway at the National Athletic Trainers’ Association (NATA) and American Physical Therapy Association (APTA) annual meetings to promote D&I to practitioners, clinicians, and scientists alike, and could offer hands-on learning labs in this space to encourage collaboration and integration of principles into clinical practice and research, culminating in a certificate for those interested. Given the strong need to convert evidence into practice in a rapidly changing clinical landscape, this approach to research and application is vital. In the simplest form, PT and/or AT focused D&I certificates would provide researchers and clinician-scientists tools and resources to effectively perform D&I research, as well as provide specific recommendations for incorporating essential topics in D&I science into their practice or research areas. There are several D&I certificate programs focused on health that can serve as a model for future certificate programs tailored to PT/AT topics.

### Add D&I to APTA and NATA research agendas and incorporate dedicated D&I programming at PT and AT professional conferences

The APTA and NATA have both created research agendas that strategically guide the growth and impact of research stemming from the respective professions (31, 32). These agendas are an indication of priorities for the organizations to focus resources and encourage growth in particular areas. Both organizations emphasize not only generating new knowledge but also ensuring that evidence translates into improved clinical practice, education, and health outcomes. Research on best practices in education and professional development are also encouraged by both organizations. D&I research priorities explicitly evaluating *how* evidence-based practices are adopted, adapted, and sustained in real-world clinical settings are needed (33). As previously mentioned, D&I is a natural fit for these objectives and specific language and D&I research priorities should be added to these agendas.

These research agendas also serve as roadmaps for professional conference programming, with specific areas of research and practice highlighted each year. These conferences should begin to include dedicated D&I programming, highlighting its direct relationship with clinical practice and research applications. Such programming would make clear to attendees D&I is necessary to bridge the gap across the various aspects of the fields and different clinicians’ and scientists’ roles. This would illustrate commitment to D&I in the fields of PT and AT as well as its importance in translation to clinical practice.

### Intentionally support and develop dedicated PT and AT D&I researchers and foster a dedicated professional network

Another approach to developing PT and AT researchers and clinician-scientists proficient in D&I is through dedicated mentoring and a dedicated professional network. For example, the NATA currently has an early-career mentoring program where more experienced faculty mentor early-career faculty, and a separate program that pairs faculty with current doctoral level students. These programs could be leveraged to provide opportunities for direct mentoring, where a dedicated number of mentor pairings could intentionally be D&I pairings. Given that D&I is broad in focus and application, there is the opportunity for a wide range of faculty to benefit from such mentorship, regardless of their specific area of emphasis. Fostering a dedicated D&I professional network within PT and AT that includes strategies to facilitate collaboration and knowledge sharing among researchers in the field can also support opportunities for researcher development and mentorship. The professional organizations of PT and AT could provide D&I resources and connections on their websites and at professional conferences to support these networks.

Fellowships dedicated to D&I in PT and AT should be developed and ultimately accredited by PT and AT credentialing bodies. A natural fit for these fellowships is in conjunction with doctoral level programs where D&I is already a focus. Fellowship opportunities can be made even more robust by partnering with institutions that have a translation science or related institute, as many of these have a D&I arm. For example, the translation science institute at the University of North Carolina-Chapel Hill has implementation science consultants as well as a full implementation science arm available for collaboration and training opportunities. Other examples can be found in Table 1, where several universities have, but may not fully leverage, clinical translational science collaborations with PT and AT faculty. These fellowships should be accredited by PT and AT bodies, lending importance and authority to this form of professional development.

Finally, organizations supporting PT and AT research should consider earmarking funding specific to D&I, given the direct need for translation to practice as well as practice-based research. Such funding is essential to provide opportunities for researchers and clinician-scientists to fill identified gaps or skill deficiencies in a systematic and deliberate manner. Taken together, intentional PT and AT researcher development increases the impact of D&I in these allied health care professions.

## Call to longer-term action

### D&I policy inclusion in CAPTE and CAATE standards

D&I science directly supports and strengthens the accreditation standards set forth by CAATE (1) and CAPTE (2). Both CAATE and CAPTE emphasize the use of EBP across multiple accreditation standards. D&I science goes beyond developing interventions to focus on how best to implement evidence-based interventions that are effective and culturally appropriate in real-world contexts. Integrating 2–3 modules on D&I foundational principles into an existing course, such as Evidence-Based Practice or MSK Evaluation, focusing on topics such as rationale for D&I practice and research, D&I terminologies, or basic D&I concepts in knowledge translation should be considered. D&I infused into a curriculum would ensure that students and practitioners not only understand EBP but are also equipped to apply it effectively in varied clinical and community settings. Furthermore, future clinicians and clinician-scientists would be trained to integrate structured D&I approaches into different areas of practice and research, such as injury prevention. For example, extensive research has been conducted in lower extremity injury prevention implementation strategies for various settings (30, 34–36). Clinicians could evaluate the need for specific strategies for groups (e.g., sports teams, occupational specialties), implement targeted strategies, and evaluate, adapt and improve efforts to maximize impact.

Both CAPTE and CAATE accreditation standards require programs to engage in ongoing assessment and continuous improvement, such as quality improvement or quality assurance projects. D&I research provides structured approaches (e.g., Plan-Do-Study-Act cycles, hybrid implementation designs) to evaluate and improve clinical processes. Integrating this content into curricula would again strengthen the skillset of future clinicians to be able to efficiently monitor, adapt, and improve their clinical practice over time.

Lastly, across all health professions even beyond PT and AT, there is an increased emphasis on interprofessional collaboration and systems-based practice. D&I research often involves studying how innovations are spread across organizations and teams, including healthcare. The principles of D&I aim to solve multi-level, complex problems that involve a variety of healthcare professions in the solution. Students learning about D&I would be more prepared to participate in a systems-wide change, working across disciplines and improving healthcare delivery on both an individual patient and macro level (see Table 2).

**Table 2:** Summary of modifications in PT and AT education and training for D&I inclusion.

Immediate actions	Intermediate actions	Longer-term actions
Integrate D&I into PT/AT curriculum	Offer D&I-specific continuing education	D&I policy inclusion in CAPTE/CAATE standards
Develop D&I post-graduate training	Incorporate D&I into APTA & NATA Research Agendas and Provide D&I Programming at Conferences	
	Intentionally support and develop dedicated D&I researchers and professional network	

## Additional considerations for the promotion of D&I in PT and AT health professions

The execution of the following considerations should also be considered by PT and AT educators, researchers, and governing bodies to incorporate D&I science in the professions.

(1) *Developing common terminology and operational definitions for D&I in PT and AT* (e.g., adherence vs. compliance; fidelity vs. quality). Differences in language and terminology often make translation across research and clinical practice difficult. Common terminology and operational definitions will help facilitate understanding and application across clinical and research continuums.
(2) *Establish a combined task force to (a) Identify who/what work is currently being done in D&I; (b) Identify opportunities to integrate D&I into existing PT/AT research strategies, and (c) Develop consensus statements.* This could be accomplished by surveys to memberships and collaboration with academic institutions where D&I work is prevalent.
(3) *Encourage editorial boards to highlight D&I work.* The British Journal of Sports Medicine featured monthly editorials/commentaries on D&I contributions; a model like this could be adopted by various PT and AT journals to underscore ongoing D&I work across professions and the evidence base supporting D&I in translating research to clinical practice.

## Discussion and concluding notes

There is a critical need to expand D&I education and practice into the fields of PT and AT. To fully embed and expand D&I into the PT and AT professions, several collaborations and alliances should be considered—between practitioners, educators, researchers and governing/licensing bodies—to foster and formalize D&I within these fields. Currently, there are numerous challenges to the successful integration of D&I science into the PT and AT professions. To our knowledge, there is currently a lack of structured/standardized curriculum in D&I science as well as a potential lack of desire to add to existing professional curriculum. However, many universities already have D&I offerings in other areas of study or within clinical translational science institutes or elective coursework. Collaboration among these professionals, along with an understanding that D&I will enhance existing curriculum should be viewed favorably, laying the groundwork for the expansion of D&I within the PT and AT professions over a longer period.

Finally, it is incumbent upon the authors in this manuscript to continue to work towards the formalization of D&I in the PT and AT profession. This advocacy should go beyond this manuscript and current roles and include leadership in the accomplishing the immediate, intermediate and longer-term actions laid out in the manuscript.

## Data Availability

The original contributions presented in the study are included in the article/supplementary material, further inquiries can be directed to the corresponding author.

## Author disclaimer

The opinions and assertions expressed herein are those of the author(s) and do not reflect the official policy or position of the Uniformed Services University or the Department of War. The contents of this publication are the sole responsibility of the author(s) and do not necessarily reflect the views, opinions or policies of The Henry M. Jackson Foundation for the Advancement of Military Medicine, Inc. Mention of trade names, commercial products, or organizations does not imply endorsement by the U.S. Government.
